# Overcoming Barriers to Heterologous Gene Expression Uncovers Regulatory Constraints in the Mucoralean Pathogen *Rhizopus delemar*

**DOI:** 10.3390/jof12100771

**Published:** 2026-10-15

**Authors:** Andrew Joseph Terrebonne, Muxing Liu, Ryan Rumsey, Tuo Wang, Ashok Aiyar, Zhengguang Zhang, Chen Ding, Ping Wang

**Affiliations:** 1 Department of Microbiology, Genetics, and Immunology, Louisiana State University Health Sciences Center, New Orleans, LA 70112, USA; 2 Department of Plant Pathology, College of Plant Protection, Nanjing Agricultural University, Nanjing 210095, China; liumuxing@njau.edu.cn (M.L.); zhgzhang@njau.edu.cn (Z.Z.); 3 Department of Chemistry, Michigan State University, East Lansing, MI 48824, USA; wangtuo1@msu.edu; 4 Department of Pharmacology, Biochemistry, and Experimental Therapeutics, Louisiana State University Health Sciences Center, New Orleans, LA 70112, USA; 5 Department of Pathogenic Microbiology, School of Basic Medical Science, China Medical University, Shenyang 110000, China; dingchen@mail.neu.edu.cn

**Keywords:** mucormycosis pathogen, regulation of gene expression, codon usage bias, targeted fluorescent protein expression

## Abstract

*Rhizopus* species are a leading cause of mucormycosis, an aggressive and life-threatening invasive fungal disease that affects immunocompromised individuals. Despite their clinical importance, mucoralean pathogens, especially *R. delemar*, are difficult to manipulate genetically because of coenocytic growth, multinucleate hyphae, difficulty with transformation, and limited selectable markers. These obstacles hinder the study of innate stress resistance and disease mechanisms. Previous efforts to express heterologous genes or implement gene-editing systems with vectors designed for model fungi have been largely unsuccessful or inefficient in *R. delemar*, suggesting unique requirements for gene expression in this organism. In this study, we tested nuclear-targeted fluorescent protein expression and identified differences in proline and arginine codon usage between heterologous and endogenous coding sequences. We then evaluated gene expression efficiency using various combinations of heterologous and endogenous regulatory elements and coding sequences, including six promoters, two terminators, three histone fusion proteins, and two fluorescent reporters, with or without an exogenous nuclear localization signal (NLS). Endogenous promoters orthologous to those with high transcriptional activity produced robust fluorescence, in contrast to weak fluorescence from constructs with heterologous sequences. Notably, fluorescence was also observed in a construct lacking an experimentally provided terminator, and the H2B-GFP fusion protein did not require an exogenous NLS for nuclear localization. These results show that efficient heterologous gene expression in *R. delemar* requires optimization at several levels, including promoter selection, coding sequence compatibility, and expression-cassette design. Although the direct impact of codon usage on expression efficiency remains to be tested, the expression systems developed here provide a platform for molecular analysis of *R. delemar* biology and pathogenesis.

## 1. Introduction

*Rhizopus* is a genus within the order Mucorales, characterized by fast-growing, coenocytic hyphae that are aseptate and multinucleate. This genus includes major causal agents of mucormycosis, such as *R*. *delemar* (formerly *R. arrhizus* var. *delemar*), *R. arrhizus* (syn. *oryzae*), *R. microsporus*, and *R. stolonifer* [1,2]. Mucormycosis is a severe invasive fungal disease, most often affecting individuals with underlying conditions, such as hematologic malignancies, prolonged neutropenia, diabetic ketoacidosis (DKA), or those receiving immunosuppressive therapies [3,4]. Current standard management relies on systemic antifungal therapy and, when feasible, surgical debridement of infected tissue [5]. However, outcomes remain poor even with aggressive interventions, particularly in patients with persistent neutropenia or disseminated infection [3,6]. Despite the substantial clinical burden of the disease, the molecular pathogenesis underlying this disease remains poorly understood, which limits progress in developing better diagnostic and therapeutic strategies.

In the United States, *R. delemar*, *R. oryzae*, and *Rhizopus microsporus* are the most frequently identified mucoralean pathogens associated with human mucormycosis (Improving clinical outcomes for Mucormycosis and other rare mold infections, NIH workshop, 2026). While genetic tools exist for some related but different *Mucor* species [7,8], molecular genetics in *Rhizopus* species remain limited. Multinucleate hyphae and spores, inefficient homologous recombination, a lack of auxotrophic markers, and intrinsic high resistance to many antifungal compounds used for selection collectively complicate genetic manipulation. Reliable expression of engineered and heterologous proteins is essential for building these resources. For example, fluorescence recovery after photobleaching (FRAP) and bimolecular fluorescence complementation (BiFC) require expression of fluorescently tagged proteins. Genome-editing systems based on transcription activator-like effector nucleases (TALENs) or clustered regularly interspaced short palindromic repeats (CRISPR)-Cas systems depend on effective expression of nuclease proteins and, for CRISPR systems, guide RNAs (gRNAs) [9,10]. Additionally, nuclear labeling could help assess nuclear distribution and guide attempts to enrich for propagules containing fewer nuclei. Thus, establishing a reliable heterologous expression system is an important step for molecular analysis in *Rhizopus*.

Few studies have previously evaluated heterologous protein expression in *Rhizopus.* For example, Tsuboi et al. used a glucoamylase A promoter to express TALEN proteins in *R. arrhizus* [9], and Mertens et al. examined cytoplasmic GFP expression in *R. delemar* using promoters associated with pyruvate decarboxylase, glucoamylase A, and phosphoglycerate kinase genes [11]. However, no systematic evaluation of endogenous and heterologous regulatory elements has been conducted in *R. delemar* or other mucoralean fungi. Here, we evaluated six promoters, two terminators, three histone proteins, two fluorescent reporters, and a nuclear localization signal (NLS) in various combinations to define factors that influence gene expression efficiency in *R. delemar*. We also identified a likely codon usage preference involving proline and arginine codons, indicating that this pattern may also limit heterologous gene expression in this fungus. This study represents the first systematic evaluation of gene expression in *R. delemar* and provides a platform to facilitate future genetic and pathogenesis studies in this organism.

## 2. Results

### 2.1. Codon Usage Analysis Reveals a Preference Involving Proline and Arginine Codons in R. delemar

Previous attempts to adapt CRISPR-Cas9 editing in *R. delemar* using the pMCas9:tRNAp-gRNA (pMCas9) vector resulted in a single-nucleotide deletion in the guide RNA target region of the *PYRF* gene [10]. pMCas9 was originally developed for the rice blast fungus *Magnaporthe oryzae*, an ascomycete, and it contains a glutaminyl (Gln)-tRNA-gRNA expression cassette [12], as well as a codon-optimized Cas9 expression cassette driven by a constitutively active translation elongation factor (Tef1) promoter [13]. Editing of additional genes with pMCas9 has proved challenging, which could be due to suboptimal expression of heterologous components, potentially arising from differences in codon usage or incompatibility of regulatory elements.

To assess the compatibility of heterologous regulatory and coding sequences, we used the pKD9-H2B-mCherry (pKD9) vector, which expresses an H2B-mCherry fusion protein under the control of the *M. oryzae* histone *H3* promoter and ribosomal protein *RP27* terminator [14]. We first examined H2B coding sequences of *R. delemar* and *M. oryzae* for potential codon bias. Using SnapGene software Version 8.2.3 (Dotmatics, available at SnapGene.com), which determines codon frequency as a relative proportion of synonymous codons and references its default standard reference tables retrieved from the Kazusa Codon Usage Database [15], we observed differences in the frequency of proline and arginine codons.

**Figure 1:**
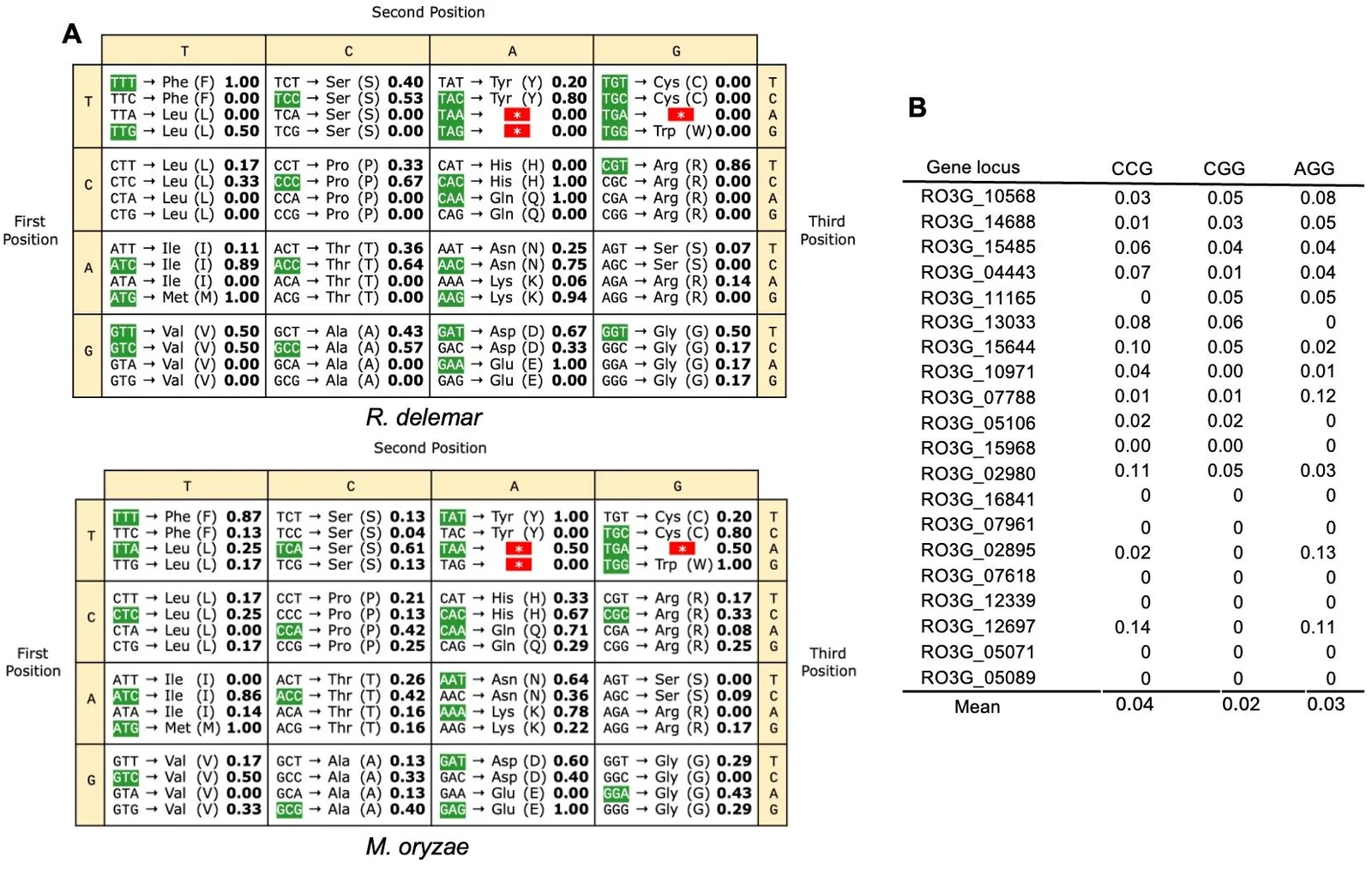
*R. delemar* exhibits codon usage bias for proline and arginine. (**A**) Comparison of H2B gene codon usage between *R. delemar* and *M. oryzae* shows differences in codon usage for proline and arginine. CCG for proline and CGG and AGG for arginine are all absent from *R. delemar* (top panel), while their frequencies in *M. oryzae* are 0.25, 0.25, and 0.17, respectively (bottom panel). (**B**) Codon usage analysis of twenty genes in *R. delemar*. Observed average frequencies of CCG, CGG, and AGG per gene are 0.04, 0.02, and 0.03, respectively. Codon usage frequency was determined as a relative proportion of synonymous codons, and a frequency value of 1 represents 100% usage of that specific codon for its corresponding amino acid. The asterisks denote stop codons.

In SnapGene’s calculation, a frequency value of 1 represents 100% usage of that specific codon for its corresponding amino acid. We found that the CCG (proline), CGG (arginine), and AGG (arginine) codons were absent from H2B (H2B1) of *R. delemar*, but occurred at frequencies of 0.25, 0.25, and 0.17 in *M. oryzae*, respectively (Figure 1A). These codons were also absent from the second H2B, H2B2, in *R. delemar*. We expanded this analysis to twenty additional genes from *R. delemar* (listed in Section 4) and found that the occurrence of CCG, CGG, and AGG codons was also very low, with frequencies of 0.04, 0.02, and 0.03, respectively (Figure 1B). Further inquiries of the Kazusa Codon Usage Database showed that the occurrences of CCG, CGG, and AGG codons are 0.02, 0.03, and 0.01 in *R. oryzae*, 0.04, 0.01, and 0.01 in *Mucor circinelloides f. lusitanicus*, and 0, 0, and 0 in *R. microsporus* [15]. In contrast, CCG, CGG, and AGG codons occur at frequencies of 0.27, 0.08, and 0.17 in *M. oryzae*, and 0.26, 0.15, and 0.13 in *Aspergillus fumigatus* Af293 [15]. The relatively infrequent use of CCG, CGG, and AGG codons in Mucorales suggests that codon usage should be carefully considered when designing expression constructs. Nevertheless, a broader analysis is needed to further define codon usage preference and clarify the impact of these patterns on gene expression.

### 2.2. Identification of Endogenous Regulatory Sequences from R. delemar

**Figure 2:**
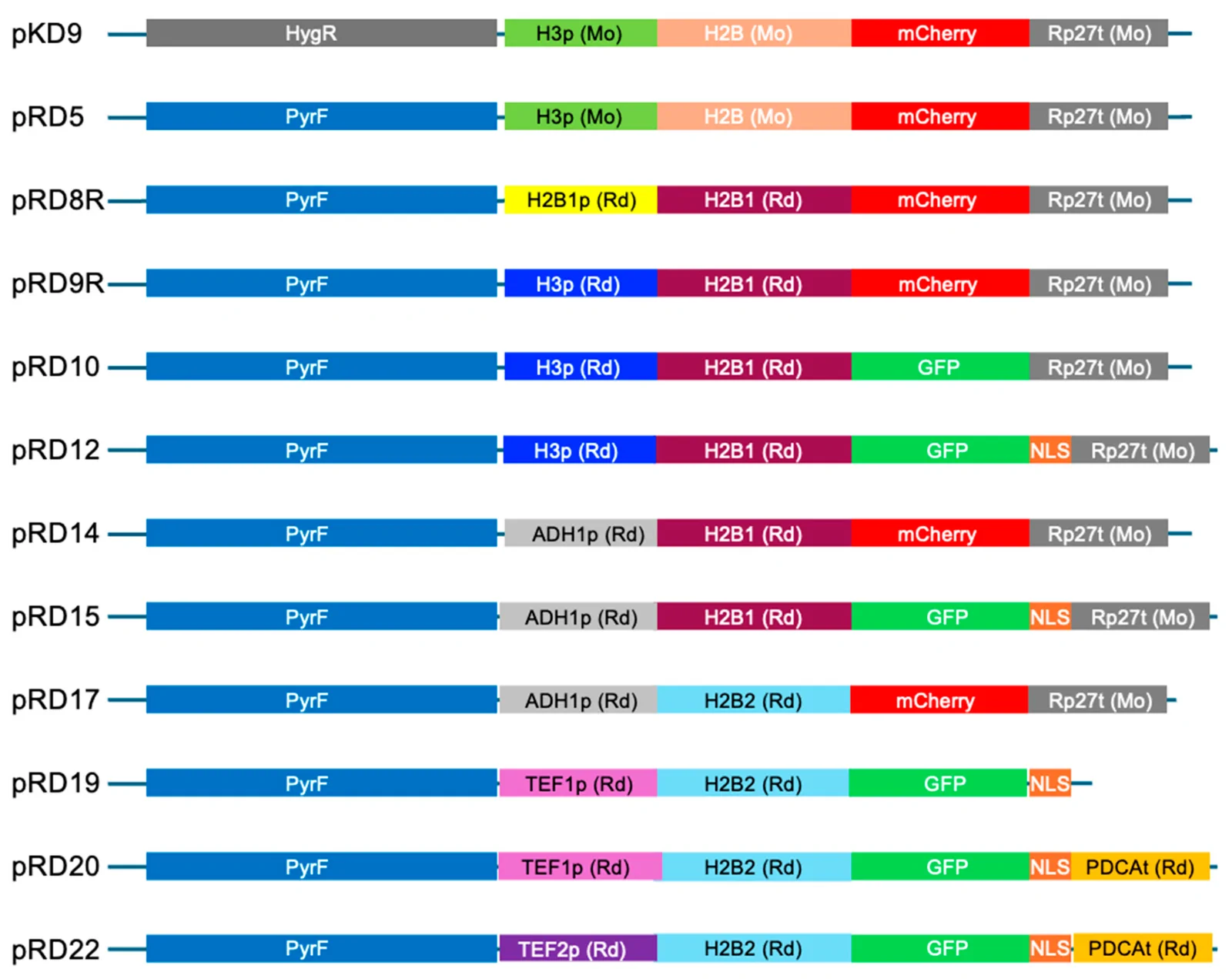
Schematic diagrams of vector designs. Plasmids pRD5-pRD17 were derived from pKD9 and contain either an H2B-mCherry or H2B-GFP fusion gene. Construction was made primarily by cloning PCR-modified DNA fragments. Plasmids pRD19-pRD22 were constructed using the MiniT cloning vector and Takara In-Fusion cloning. pRD8R contains the *H2B1* promoter (*H2B1p*); pRD9R-pRD12 contain the *H3* promoter (*H3p*); pRD14-pRD17 contain the *ADH1* promoter (*ADH1p*); pRD19 and pRD20 contain the *TEF1* promoter (*TEF1p*); and pRD22 contains the *TEF2* promoter (*TEF2p*). pRD8R-pRD15 contain H2B1, whereas pRD17-pRD22 contain H2B2. pRD12, pRD15, pRD19, pRD20, and pRD22 include a nuclear localization signal (NLS). pRD19 lacks a terminator.

To evaluate heterologous reporter expression, we modified pKD9 for selection in *R. delemar*. Because *R. delemar* can grow in the presence of 1 mg/mL hygromycin B, the hygromycin B-resistance cassette (*HYGB*) was replaced with a *PYRF* cassette conferring uracil prototrophy [10,16]. The resulting vector, pRD5 (Figure 2), was introduced into the *pyrF* auxotrophic strain f1 by biolistic transformation [10]. Transformants were selected and subcultured five times on synthetic medium lacking uracil. The presence of the expression cassette was confirmed by PCR and sequencing of the resulting amplicons using primers listed in Table S1.

**Figure 3:**
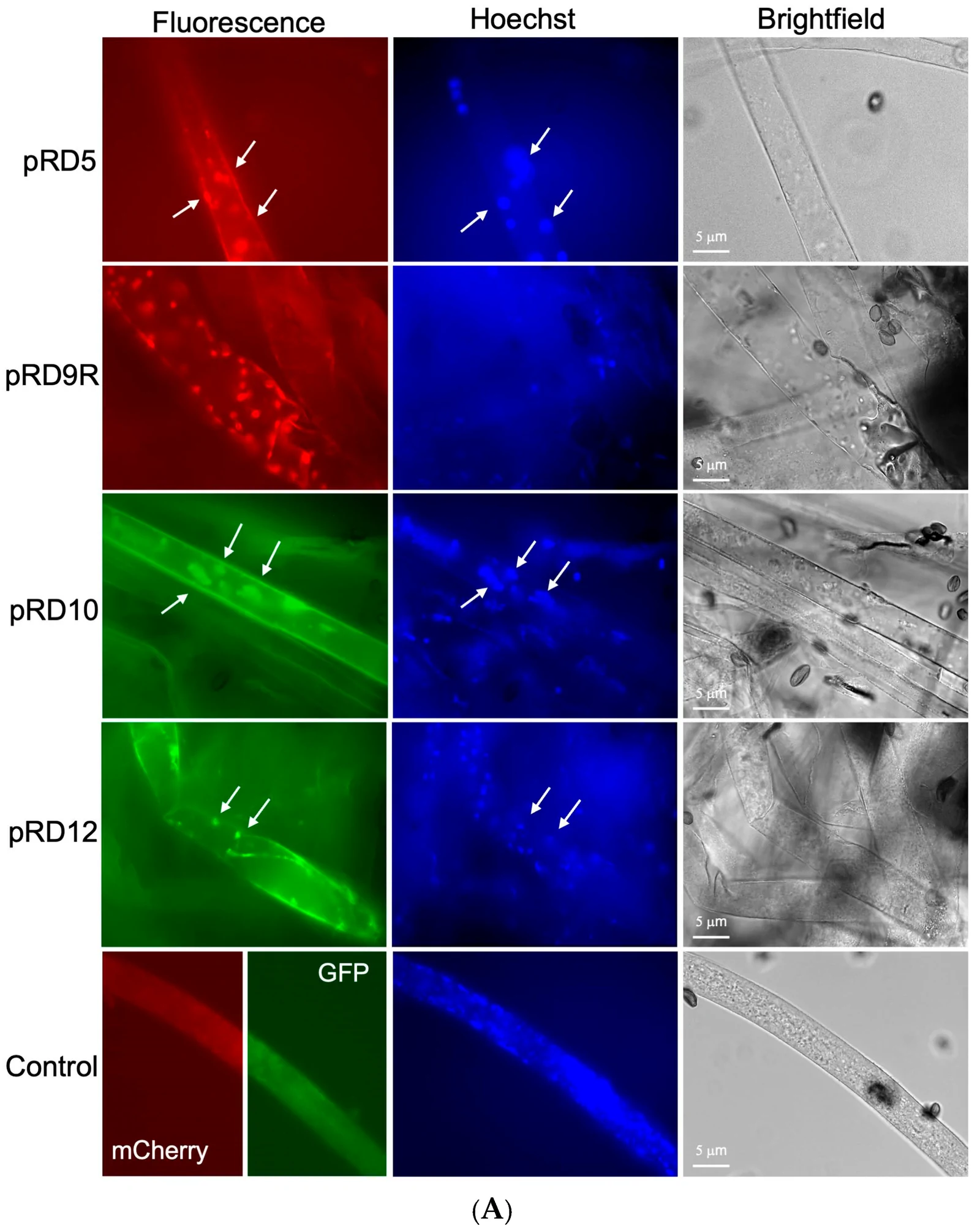

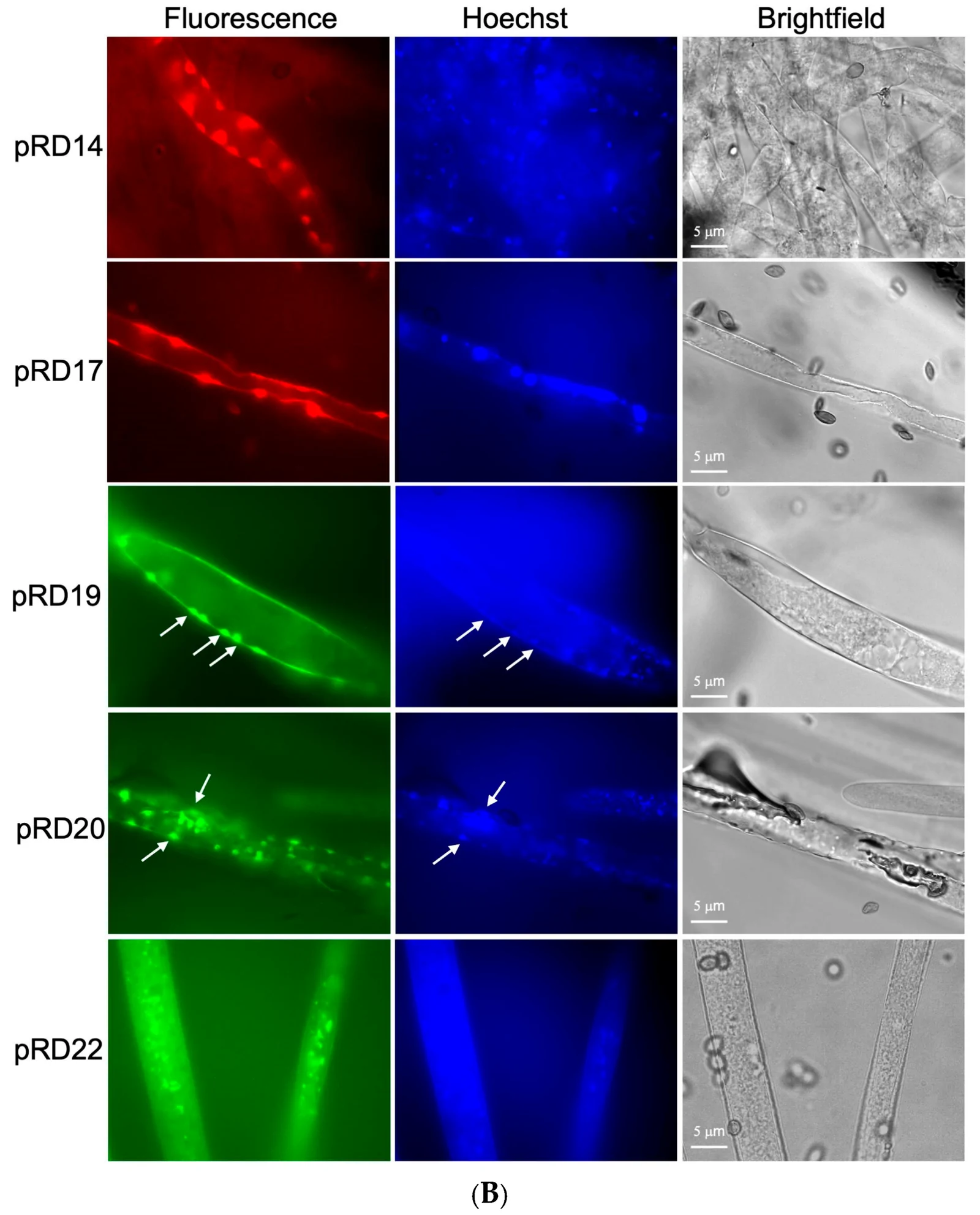
Microscopic examination of nuclear fluorescent reporters in *R. delemar*. Transformants were subcultured at least five times on SD-Ura medium before analysis. For microscopy, transformants were cultured for 2 days at 30 °C. Hyphal and sporangiophore sections were placed on microscope slides with 15 μL of mounting medium containing 90% glycerol (*v*/*v*) and 5 μg/mL Hoechst 33342 in 1× PBS. Brightfield and fluorescence images were acquired using a Zeiss Imager A2 microscope (Carl Zeiss Microscopy, LLC, White Plains, NY, USA) equipped with an HXP 120 illumination system. Images were processed using Adobe Photoshop 2026. (**A**) Transformants of pRD5, pRD9R, pRD10, pRD12, and reference strain FGSC-9543 control. (**B**) Transformants of pRD14, pRD17, pRD19, pRD20, and pRD22. The first column shows reporter fluorescence, the second column shows Hoechst fluorescence, and the third column shows brightfield. These images depict the same Hoechst-stained specimen acquired through different fluorescence filter sets and they do not represent samples with and without Hoechst. Arrows indicate examples of reporter fluorescence that appears spatially coincident with Hoechst-stained structures.

Nuclear fluorescence was observed through microscopy and digital image acquisition in sporangiophores (specialized hyphae) of pRD5 transformants (Figure 3A); however, the fluorescence signal was weak, in contrast to robust fluorescence expression reported in *M. oryzae* and the entomopathogenic fungus *Trichophyton mentagrophytes* [14,17]. This result suggests that heterologous regulatory and coding sequences can support H2B-mCherry expression in *R. delemar*, but expression was likely inefficient.

We then tested whether endogenous regulatory and coding sequences could improve fluorescence expression. Promoters, terminators, and histone sequences were identified from the genome of the reference strain FGSC-9543, and constructs were designed to compare endogenous and heterologous components. GFP was also included as an alternative reporter because its coding sequence contains few of the infrequently used proline and arginine codons identified above.

Several regulatory sequences were previously identified that associate with genes encoding pyruvate decarboxylase (*PDCA*), glucoamylase A (*AMYA*), phosphoglycerate kinase (*PGK1*), and lactate dehydrogenases (*LDHA* and *LDHB*) in *R. delemar* and *R. arrhizus* (*R. oryzae*). These promoters differed in their ability to support gene expression [11,18,19]. We isolated *PDCA*, *AMYA*, and *PGK1* genes by PCR amplification from strain FGSC-9543, but not *LDHA* and *LDHB*, which were only found in *R. arrhizus* [18]. To compare endogenous promoters expected to differ in strength, we selected genes encoding histone H2B1 (*H2B1*, XP_067527132.1), H2B2 (*H2B2*, KAG1440616.1), H3 (*H3*, XM_067658520.1), alcohol dehydrogenase homolog Adh1 (*ADH1*, XP_067516736), and translation elongation factor homologs Tef1 (*TEF1*, XM_067663078) and Tef2 (*TEF2*, XM_067655654), and isolated their predicted promoter regions. We also cloned the endogenous terminator associated with the pyruvate decarboxylase gene *PDCAt* (AF282846). We evaluated these endogenous regulatory elements alongside *M. oryzae H3p* and the ribosomal protein Rp27 terminator (*RP27t*) from pKD9. The resulting vector collection enables us to compare the effects of promoter origin and predicted strength, histone fusion partner, fluorescent reporter, terminator identity, and nuclear localization signal (NLS) on expression in *R. delemar*. Regulatory sequences endogenous to *R. delemar* are provided in Table S2.

Because *R. delemar* forms coenocytic, multinucleate hyphae, nuclear labeling could facilitate analysis of nuclear distribution, inheritance, and dynamics, and could help to isolate or enrich uninucleate cells, an insurmountable bottleneck in genetic analysis of this fungus. The endogenous H2B1 and H2B2 proteins were fused to mCherry or GFP. In selected mCherry and GFP constructs, the simian virus 40 NLS, PKKKRKV, was also added to the C-terminus of the construct to determine whether it enhances nuclear targeting. Constructs containing either endogenous *PDCAt* or exogenous *RP27t*, as well as a construct lacking an experimentally supplied terminator, were also evaluated.

### 2.3. Construction of Vectors for Nuclear-Targeted Expression of mCherry and GFP

To test whether endogenous regulatory and structural sequences would improve fluorescence expression, *R. delemar* H2B1 and H3 promoters and one of the H2B histones, H2B1, were used to replace the corresponding components in pRD5. This generated pRD8R, containing *H2B1p-H2B1-mCherry-RP27t*, and pRD9R, containing *H3p-H2B1-mCherry-RP27t*. The GFP coding sequence containing few of the infrequently used codons identified in *R. delemar* was also used as an alternative reporter. This generated pRD10 (*H3p-H2B1-GFP-RP27t*). Addition of NLS to the C-terminus of *GFP* in pRD10 resulted in pRD12 (*H3p-H2B1-GFP-NLS-RP27t*).

The Adh1 promoter is conserved, moderately strong, and inducible. Using the endogenous *ADH1p*, we constructed pRD14 (*ADH1p-H2B1-mCherry-RP27t*), pRD15 (*ADH1p-H2B1-GFP-NLS-RP27t*), and pRD17 (*ADH1p-H2B2-mCherry-RP27t*). Finally, we identified and incorporated the Tef1 and Tef2 promoters orthologous to well-documented Tef1 and Tef2 in budding yeast (*Saccharomyces cerevisiae*) and mammalian cells to yield pRD19 (*TEF1p-H2B2-GFP-NLS*), pRD20 (*TEF1p-H2B2-GFP-NLS-PDCAt*), and pRD22 (*TEF2p-H2B2-GFP-NLS-PDCAt*). pRD19 lacked a terminator. pRD17, pRD19, pRD20, and pRD22 all contain a second H2B histone protein, H2B2; pRD20 and pRD22 additionally contain the endogenous *PDCA* terminator. Vector designs are illustrated in Figure 2.

### 2.4. Endogenous Promoters Enhance Nuclear Fluorescent Protein Expression in R. delemar

Twelve expression constructs containing various combinations of promoters, histones, fluorescent proteins, terminators, and an NLS were generated. Each vector was introduced into the *pyrF* auxotrophic mutant f1 by biolistic transformation using either circular or linearized plasmids. Transformants were subcultured at least five times (ten times for pRD14, pRD19, and pRD22) by transferring hyphal branches, and the presence of the expression-cassette sequences was verified by PCR using primers spanning the promoter-H2B1 or H2B1/H2B2–fluorescent protein junctions (Figure S1).

Fluorescence was examined using a Zeiss Axio Imager A2 fluorescence microscope (Carl Zeiss Microscopy, LLC, White Plains, NY, USA). As noted earlier, pRD5 transformants displayed weak fluorescence in sporangiophores, and punctate fluorescence appeared spatially coincident with Hoechst-stained structures, consistent with the nuclear localization of H2B-mCherry (Figure 3A). However, the two signals did not overlap completely, which could be due to variations in hyphal pigmentation and permeability to the Hoechst stain. Patchy fluorescence could reflect the heterokaryotic state of the transformants despite repeated subculturing. Replacement with endogenous *H2B1p* and *H3p* supported fluorescence expression. Few fluorescent structures were observed in pRD8R transformants, whereas pRD9R transformants showed more fluorescence in structures mimicking nuclei (pRD9R shown in Figure 3A), suggesting that H2B1p and H3p may support reporter gene expression differently under the conditions examined. Transformants carrying pRD10 and pRD12 also displayed GFP fluorescence (Figure 3A), indicating GFP as a good reporter. The lack of an NLS sequence did not prevent nuclear localization of the fusion protein, indicating that the H2B-fusion is sufficient for nuclear localization.

The Adh1 promoter (*ADH1p*) allowed robust fluorescence expression in transformants carrying pRD14, pRD15, and pRD17 (pRD14 and pRD17 shown in Figure 3B). pRD17 also contains a second H2B protein, H2B2, and its transformants showed fluorescence patterns similar to those harboring pRD14 (Figure 3B). Fluorescent nuclei were also apparent in transformants carrying *TEF1p*- and *TEF2p*-based constructs, including pRD19, pRD20, and pRD22 (Figure 3B). pRD19 showed fluorescence in the absence of an experimentally provided terminator under the conditions tested. Robust fluorescence in transformants harboring pRd14, pRD19, pRD20, and pRD22 suggests the endogenous *ADH1p*, *TEF1p*, and *TEF2p* as effective promoters of *R. delemar*.

We also examined the stability of fluorescence expression in selected transformants. pRD14, pRD19, and pRD22 transformants were subjected to five additional rounds of hyphae branch transfer (ten rounds in total), and all subcultures retained fluorescence. Fluorescence was generally visible after 2–3 days of growth at 30 °C on either synthetic defined medium lacking uracil (SD-Ura) or semi-defined potato dextrose agar (PDA).

### 2.5. Quantitative RT-PCR Confirms Fluorescence Reporter Expression

**Figure 4:**
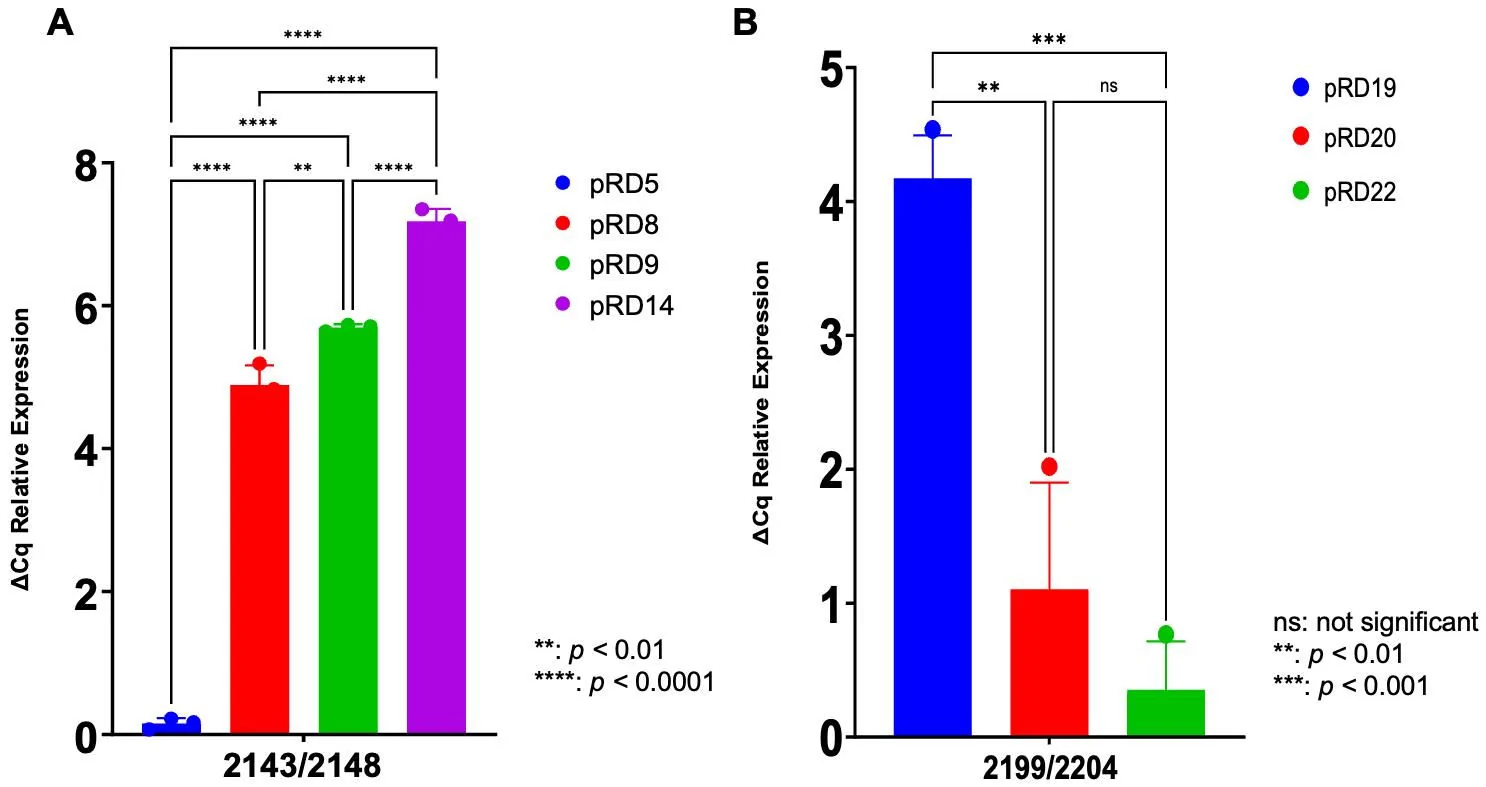
Quantitative RT-PCR analysis of fluorescent protein transcript levels in *R. delemar* transformants. Relative transcript levels of mCherry (**A**) and GFP (**B**) in the indicated transformants. mCherry was amplified using primer pair PW2143/PW2148, and GFP was amplified using primer pair PW2199/PW2204. Transcript levels were normalized to those of *R. delemar ACT1* (mCherry) and *PYRF* (GFP), amplified using primer pairs PW2522/PW2523 and PW2293/PW2297, respectively. Statistical analysis was performed using ordinary one-way ANOVA with Tukey’s test after confirming normal distribution with the Shapiro–Wilk test, as described in Section 4. Each biological sample includes three experimental replicates, and the error bars indicate the variability, expressed as standard deviation (SD). **: *p* < 0.01. ****: *p* < 0.0001.

Microscopic examination showed that fluorescence levels varied among transformants carrying different vector constructs. The detection of fluorescence in transformants containing pRD5, pRD8R, pRD9R, pRD10, and pRD12 required assistance from digital image acquisition (Zeiss, Carl Zeiss Microscopy, LLC, White Plains, NY, USA), whereas fluorescence in the remaining transformants was readily detectable directly through the microscope eyepieces. To substantiate the expression of fluorescent transcripts, reverse transcription quantitative PCR (qRT-PCR) was performed for selected transformants. Transformants carrying pRD5 showed low levels of *mCherry* transcripts, whereas pRD8R, pRD9R, and pRD14 transformants showed a relatively high abundance of mCherry transcripts (*p* < 0.0001) (Figure 4A). *GFP* transcript levels were also detected at a higher level in the transformant carrying pRD19 than in those carrying pRD20 and pRD22 (*p* < 0.001). The reason for this remains unclear (Figure 4B). Despite this discrepancy, these transcript estimates were broadly consistent with the microscopy observations.

Together, these results show that both heterologous regulatory and coding sequences can function in *R. delemar*, but their expression efficiency is low. Regulatory sequences endogenous to *R. delemar* provide robust expression efficiency. Among the endogenous promoters examined, *H2Bp* and *H3p* induce gene expression, whereas *ADH1p*, *TEF1p*, and *TEF2p* support more robust expression.

## 3. Discussion

Invasive mucormycosis predominantly affects individuals with compromised immunity. Among members of the order Mucorales, *Rhizopus* species are the most common etiological agents. Although species such as *R. oryzae* have been exploited for industrial fermentation, molecular genetic analysis of the pathogenic species, including *R. delemar*, remains limited. To improve *R. delemar* tractability, we systematically evaluated components of heterologous expression cassettes for gene expression. Fluorescent protein expression targeted to nuclei was achieved using a vector originally developed for *M. oryzae*, an ascomycete fungus, demonstrating that heterologous regulatory elements can function across substantial phylogenetic distances. However, the expression efficiency was low. Endogenous promoters generally improved expression with *TEF1p* and *TEF2p* endogenous to *R. delemar* producing robust and broadly distributed nuclear fluorescence. The improved performance of endogenous promoters likely reflects their conserved function and greater compatibility with the transcriptional machinery of *R. delemar*.

Differences in core promoter architecture, transcription-factor recognition, nucleosome positioning, or chromatin accessibility could reduce the activity of promoters derived from *M. oryzae*. Such incompatibility may also have contributed to the limited efficiency of earlier CRISPR-Cas9 experiments using expression components optimized for ascomycete fungi. Nevertheless, because promoter identity was not tested independently of all other construct variables in every comparison, the relative contributions of promoter sequence, the identity of fluorescent reporter protein, histone fusion partner, and cassette architecture will require further analysis.

We also identified a codon usage preference involving several proline- and arginine-encoding codons in genes of *R. delemar*. This finding is consistent with an earlier codon usage survey of the Mucorales fungi. Still, additional surveys are needed to determine whether this trait is broadly applicable to all mucoralean fungi. Codon usage can influence translation through differences in tRNA abundance, mRNA stability, protein elongation, and protein folding. Infrequently used codons in heterologous coding sequences may therefore contribute to inefficient protein production in *R. delemar*. The current study establishes an association, not a direct causal relationship, between codon usage and expression efficiency. A direct comparison of otherwise identical native and codon-optimized coding sequences is necessary to determine whether codon optimization improves expression.

Fusion of fluorescent reporters to H2Bs was sufficient to direct fluorescence to nuclei. Histones are naturally imported into the nucleus, and the H2B component of the fusion proteins therefore appears sufficient to mediate nuclear targeting. This observation simplifies future nuclear-labeling constructs by eliminating the need for an additional heterologous localization sequence. Additionally, pRD19 lacking an experimental terminator produced detectable GFP expression, indicating that omitting a defined terminator sequence did not adversely affect protein expression. Transcription may have terminated through cryptic downstream sequences in the plasmid backbone, or RNA processing may have occurred at an uncharacterized sequence. Direct measurement of transcript ends and comparisons among otherwise identical constructs are needed to determine how transcription terminates in this system.

Fluorescence expression remained patchy in the transformants we examined. Several basic characteristics of *R. delemar* could contribute to this pattern. Biolistic transformation of multinucleate cells likely produces heterokaryotic transformants containing both transformed and untransformed nuclei. Unequal nuclear segregation during hyphal growth could then generate regions with different proportions of transformed nuclei. Variation in transgene copy number, transcriptional state, or epigenetic silencing could further contribute to the patchy pattern observed in these transformants. We have performed multiple and repeated hyphal branch subculture transfers; however, continued single hyphal tip transfer to further reduce heterogeneous nuclei might help resolve the basis of this heterogeneity.

Finally, the qRT-PCR results indicate differences in mCherry transcript abundance among the tested constructs. The heterologous *H3p* produced weak expression, whereas the constructs fused with endogenous promoters supported better expression. Since endogenous promoter constructs also differ in reporter cassette, histone sequence, NLS status, terminator identity, and vector backbone, future studies using matched expression cassettes will be important for quantitatively isolating the contribution of each component.

Collectively, our findings suggest that efficient heterologous gene expression in *R. delemar* is constrained at multiple regulatory levels. These include transcriptional compatibility between promoters and the endogenous transcription machinery, translational efficiency potentially influenced by codon usage, and expression-cassette architecture. At the same time, contributions of certain components commonly incorporated into fungal expression vectors, such as an exogenous NLS or a defined transcriptional terminator, were not apparent under the conditions we examined. The expression platform established here provides a molecular toolkit for *R. delemar* and should facilitate future applications involving fluorescent-protein tagging, protein-localization studies, gene complementation, and genome editing. Importantly, these tools will support mechanistic investigations of biology and virulence in *R. delemar*.

## 4. Materials and Methods

### 4.1. Fungal Strains, Plasmids, and Primers

The *Rhizopus delemar pyrF* auxotrophic strain f1, derived from the reference strain FGSC-9543, was used as the recipient strain for all transformation experiments [10]. Cultures were routinely maintained on potato dextrose agar (PDA) and propagated on PDA+ medium (PDA supplemented with 1% yeast extract, 1% peptone, and 1% glycerol) for spore production. Genetic transformation was performed using MMC-Ura medium (1% Casamino Acids, 0.05% yeast nitrogen base without amino acids or ammonium sulfate, 0.05% yeast dropout supplement lacking Uracil, 2% glucose, pH 4.5) supplemented with 0.5 M sorbitol. Media preparation, culture conditions, and transformation procedures were the same as those previously described [10].

The plasmid pKD9 was constructed for nuclear expression of fluorescence in the rice blast fungus *M. oryzae* [14]. The *PYRF* marker gene was described previously [16]. Oligonucleotide primers used in this study are listed in Table S1. PCR products were initially cloned into pCR2.1 (Invitrogen, Thermo Fisher Scientific, Waltham, MA, USA), pDrive (Qiagen, Germantown, Maryland, USA), or MiniT (New England Biolabs, Ipswich, MA, USA) vectors and verified by Sanger sequencing before any downstream application.

### 4.2. Analysis of Codon Usage Frequency

*R. delemar* (FGSC-9543) coding sequences of twenty genes, not including *H2B1* and *H2B2*, were selected from GenBank, and codon frequencies were individually examined using SnapGene software Version 8.2.3 (Dotmatics). We analyzed codon usage frequencies using SnapGene software, which determines codon frequency as a relative proportion of synonymous codons, and a frequency value of 1 represents 100% usage of that specific codon for its corresponding amino acid. The selected genes represent those from initial sequence annotation by Ma and colleagues [20] and gene homologs encoding acyl-CoA desaturases, glucan synthase, and multidrug resistance pumps (Wang, unpublished data). Gene identifiers are RO3G_10568, RO3G_14688, RO3G_15485, RO3G_04443, RO3G_11165, RO3G_13033, RO3G_15644, RO3G_10971, RO3G_07788, RO3G_05106, RO3G_15968, RO3G_02980, RO3G_16841, RO3G_07961, RO3G_02895, RO3G_07618, RO3G_12339, RO3G_12697, RO3G_05071, and RO3G_05089.

### 4.3. Construction of Fluorescent Protein Expression Vectors

#### 4.3.1. Construction of pRD5, pRD8R, and pRD9R

To generate pRD5, the *PYRF* gene was amplified from plasmid pSL22 [16] using primers PW2159 and PW2050. The sequence-verified fragment was excised with *Eco* RI and *Xho* I and ligated into the corresponding sites of pKD9 after removal of the hygromycin-resistance HgyB cassette. For construction of pRD8R, the *PYRF* fragment was amplified with primers PW2159/PW2170, and the *H2B1p* and *H2B1* coding sequences were amplified with primers PW2169/PW2164. The two PCR fragments were joined by overlap PCR using primers PW2159 and PW2164. The resulting *PYRF*-*H2B1p-H2B1* fragment was digested with *Eco* RI and *Sma* I and inserted into pKD9 digested with the same enzymes. For pRD9R, *PYRF*, the *H3p*, and *H2B1* were amplified using primer pairs PW2159/PW2166, PW2165/PW2168, and PW2167/PW2164, respectively. The three fragments were assembled by overlap PCR with primers PW2159 and PW2164 and cloned into the pKD9 backbone as described for pRD8R.

#### 4.3.2. Construction of GFP and ADH1 Promoter Derivative Vectors

The GFP coding sequence was amplified from *GFP-WSP1* [21] using primers PW2186 and PW2187. Following digestion with *Sma* I and *Sal* I, the *GFP* fragment replaced the *mCherry* coding sequence in pRD9R to generate pRD10. To construct pRD12, the SV40 NLS (PKKKRKV) was incorporated at the C-terminus of *GFP* in pRD10 by PCR amplification using primers PW2186 and PW2107. For pRD14, the *ADH1p* and *H2B1* coding sequences were amplified using primer pairs PW2211/PW2213 and PW2212/PW2177, respectively. The fragments were joined by overlap PCR using primers PW2211 and PW2177 and the *Eco* RI and *Sma* I digested *ADH1p*-*H2B1* fragment replaced *H3p-H2B1* of pRD9R resulting in pRD14. The same *ADH1p*-*H2B1* fragment was inserted into pRD12 to generate pRD15. In pRD17, *H2B1* of pRD14 was replaced with *H2B2*. The *ADH1p* and *H2B2* coding sequences were amplified using primer pairs PW2211/PW2242 and PW2241/PW2245, respectively, and joined by overlap PCR using primers PW2211 and PW2245.

#### 4.3.3. Construction of TEF Promoter Vectors

Unlike earlier pRD vectors with the pRD5 backbone, pRD19, pRD20, and pRD22 were constructed in the MiniT-based vector. For pRD19, the *TEF1p* and *H2B2* coding regions were amplified using primer pairs PW2302/PW2386 and PW2385/PW2207, respectively. The two fragments were joined by overlap PCR using primers PW2302 and PW2207 and cloned into MiniT. The *PYRF* selectable marker was amplified using primers PW2393 and PW2394 and inserted into the *Bam* HI site by Takara In-Fusion cloning. To construct pRD20, the endogenous *PDCAt* was amplified using primers PW2478 and PW2479, digested with *Xba* I and *Not* I, and inserted into the corresponding sites of pRD19. For pRD22, the *TEF2p* was amplified using primers PW2474 and PW2475, whereas the *H2B2-GFP-NLS-PDCAt* fragment was amplified using primers PW2476 and PW2477. The two fragments were linked by In-Fusion cloning to generate the final pRD22 construct.

Promoter sequences endogenous to *R. delemar*, including *H2B1p*, *H3p*, *ADH1p*, *TEF1p*, and *TEF2p*, are listed in Table S2. All plasmid constructs were verified by restriction analysis and nanopore sequencing before any downstream application.

### 4.4. Transformation, Transformant Verification, and Fluorescence Microscopy

Plasmid constructs were introduced into the *R. delemar pyrF* auxotrophic strain f1 by biolistic transformation as previously described [10]. Transformants were selected on uracil-deficient synthetic medium, subcultured. The presence of the expression cassette was verified by PCR amplification using construct-specific primers (Table S1).

Normally, ten verified transformants per plasmid DNA were maintained, four underwent hyphal branch transfer. For fluorescence microscopy, we cultured these four transformants independently on SD-Ura agar plates for approximately 48 h. Hyphae were mounted in 15 μL solution containing 1× phosphate-buffered saline (PBS), 90% glycerol (*v*/*v*), and 5 μg/mL Hoechst 33342 (Sigma-Aldrich, St. Louis, MO, USA). Fluorescence and brightfield images were acquired using a Zeiss Axio Imager A2 fluorescence microscope (Carl Zeiss Microscopy, LLC, White Plains, NY, USA) with an HXP 120 illumination system, a Plan-Apochromat 63x oil-immersion objective (numerical aperture 1.4), and an AxioCam digital camera (Zeiss, Carl Zeiss Microscopy, LLC, White Plains, NY, USA). The turret position, fluorophore, excitation and emission were, respectively, #2/Hoechst/365 nm/445/50 nm, #3/FITC/450–490 nm/515–565 nm, and #4/TRITC/546/12 nm/575–640 nm. Images were processed using Adobe Photoshop 2026, and adjustments were limited to brightness and contrast.

### 4.5. RNA Isolation and Quantitative RT-PCR

RNA was extracted from various types of transformants expressing mCherry or GFP fluorescent protein using the Quick-RNA Fungal/Bacterial Miniprep Kit (R2014, Zymo Research, Irvine, CA, USA). Two micrograms of total RNA from each sample were reverse-transcribed using the High-Capacity cDNA Reverse Transcription Kit (4368814, Applied Biosystems, Foster City, CA, USA).

qRT-PCR was performed using Fast SYBR Green Master Mix (Applied Biosystems) in 10-µL reaction volumes on a Bio-Rad real-time PCR system with gene-specific primers (Table S1). PCR conditions consisted of 50 °C for 2 min, 95 °C for 5 min, followed by 40 cycles of 95 °C for 3 s and 60 °C for 30 s. Fluorescence was measured at the end of each amplification cycle. Amplification specificity was verified by agarose gel electrophoresis.

The analysis included three independently cultures grown from one of the representing transformants per vector DNA, each independently culture with three technical replicates. Cq values from the three technical replicates were averaged to yield a single mean. Relative gene expression was calculated using the ∆Cq method (∆Cq = Cq target − Cq reference), normalizing the target gene to the endogenous reference genes (*ACT1* for mCherry samples, *PYRF* for GFP samples), amplified by PCR with primers PW2522/PW2523 and PW2293/PW2297. Statistical analysis was performed using ordinary one-way ANOVA with Tukey’s test after confirming normal distribution with the Shapiro–Wilk test.

## Data Availability

The data presented in this study are available on request from the corresponding author.

## Supplementary Materials

The following supporting information can be downloaded at: https://www.mdpi.com/article/10.3390/jof12100771/s1, Figure S1: PCR-based genotypic verification of selected pRD transformants; Table S1: DNA primers used in this study; Table S2: Endogenous regulatory elements used in this study.
