# Supplementary material for: Overcoming Barriers to Heterologous Gene Expression Uncovers Regulatory Constraints in the Mucoralean Pathogen Rhizopus delemar
Source: J Fungi (Basel). 2026 Oct 15;12(10):771. doi: 10.3390/jof12100771 (PMC13706632; doi:10.3390/jof12100771)

Table S1. Primers used in this study.

Primer Name	Sequence (5’-3’)	Remarks
**PW2159**	ACTCGAGTCTGAGGGAGGTATGCAAGG	Link PYRF-H3p-H2B1
**PW2050**	TCAATGAATGCCAACCTCAA	PYRF reverse
**PW2170**	GTAAAATTCTATTTTTTATTGAATTCTCAATG AATGCCAACCTC	link PYRF with H2B1 reverse
**PW2169**	GAGGTTGGCATTCATTGAGAATTCAATAAA AAATAGAATTTTAC	link PYRF with H2B1 forward
**PW2164**	TCCCGGGTTATTTGGAACTGGTGTACTTG	Link PYRF-H3p-H2B1
**PW2166**	TGGTGCTAAGCGTCACAGAAAGAATTCTCA ATGAATGCCAACCTCA	H2B1 end reverse (SmaI)
**PW2165**	TGAGGTTGGCATTCATTGAGAATTCTTTCT GTGACGCTTAGCACCA	link PYRF with H3P forward
**PW2168**	AGTAGCAGTTTCAGCAGCCATATTTAATGTT TGATATAGAAA	link H3P w/ H2B reverse
**PW2167**	TTTCTATATCAAACATTAAATATGGCTGCTG AAACTGCTACT	link H3P w/ H2B forward
**PW2186**	ACCCGGGATGGTGAGCAAGGGCGAGGAG	GFP (SmaI) forward
**PW2187**	ATCTAGAGTCGACTTA AGCGGCGGACTTGTACAGC	GFP (XbaI / SalI) reverse
**PW2107**	GGGGATCGATGCCAAGTTAGCTGGTAACC ACGTGATCGG	PYRF 'flank 5' with ClaI site added
**PW2211**	AACTCGAGATTATTATTAGAGGGAAAAAAAA GA	ADH1p forward
**PW2213**	CTTAGTAGCAGTTTCAGCAGCCATTTTTGT TATTTATTTGTATTAATTGAT	ADH1p reverse with H2B1
**PW2212**	ATCAATTAATACAAATAAATAACAAAAATGG CTGCTGAAACTGCTACTAAG	ADH1p forward with H2B1
**PW2177**	AACCCGGGTTTGGAACTGGTGTACTTGGT G	H2B1 reverse SmaI
**PW2242**	AGCAGAGGCGGATTCTTTGGGAGCCATTT TTGTTATTTATTTGTATTAATTGAT	ADH1p-H2B1 junction reverse
**PW2241**	ATCAATTAATACAAATAAATAACAAAAATGG CTCCCAAAGAATCCGCCTCTGCT	ADH1P-new H2B1 junction forward
**PW2245**	ACCCGGGTTTGGAGCTGGTGTACTTGGTG ACAG	New H2B1 SmaI
**PW2302**	AGAATTCAATTGTGTGAAAGTGTAAATTCC A	TEF1P EcoRI forward

**PW2386**	ACTTTGCAGCAGAGGCGGATTCTTTGGGA GCCATGCTATGATGAATAGAATTAGTACATG TTGGAT	TEF1P-new H2B2 junction reverse extended
**PW2385**	ATCCAACATGTACTAATTCTATTCATCATAG CATGGCTCCCAAAGAATCCGCCTCTGCTG CAAAGT	TEF1P-new H2B2 junction forward extended
**PW2207**	TTCTAGATTAAACCTTACGCTTCTTCTTAGG AGCGGCGGACTTGTACAGCTCGTCCAT	GFP 3 PRd10
**PW2393**	TGTATCGCTCGAGGGATCTCTGAGGGAGG TATGC	PYRF-GFP 5' Infusion BamHI
**PW2394**	ACCTCCTGAATTCGGATCTCAATGAATGCC AACCTCAAAA	PYRF-GFP 3'
**PW2478**	AATCTAGACTAGAGTCGACCTGCAGGAG	XbaI PDCAt F forward 3' to GFP
**PW2479**	ATGCGGCCGCATGATTACGCCAAGCTTGG A	NotI PDCAt reverse
**PW2474**	ACCATGATAGAATTCTTAAAACAAAAAAAAA GAGTAATCGATTACTAAACC	TEF2p for infusion #1 swapping forward
**PW2475**	GGCGGATTCTTTGGGTGCATTCATATCTCG ATCAAGTTCT	TEF2p promoter for infusion #1 swapping reverse
**PW2476**	CGAGATATGAATGCACCCAAAGAATCCGCC TCTG	infusion #1 vector for above swapping forward
**PW2477**	AAGAATTCTATCATGGTTTTTACCTCCTGAA	infusion #1 vector for above swapping reverse
**PW2522**	ACTGAAGCACCTTTGAAC	Actin 5' short piece for qPCR
**PW2523**	TCACGACCGGCCATATCC	Actin 3' short piece for qPCR
**PW2112**	AAACTTGAAGGGCAAGGTGGTCGT	gDNA PCR orthogonal verification
**PW2204**	CACGCTTCTCGTTAGGGTCTTTGCTCAGG GCGGACTGGGTGCTCAGGTAGTGGTTGTC AGGCAGCAGCACAGGGCCGTCGCCGATA GGGG	
**PW2334**	ATCCAACATGTACTAATTCTATTCATCATAG CATGGCTCCCAAAGAATCCGCCTCTGCT	
**PW2201**	ACCCGGGATGGTGAGCAAGGGCGAGGAG CTGTTCACCGGGGTGGTGCCTATCCTG	
**PW2556**	CAGGTCGACTCTAGTCTAGA	
**PW2199**	CTGAAGGGCATCGACTTCAAGG	qPCR
**PW2143**	AGGTGGCAATATCGAGGTGGGCTTCTCCG GGAATCGAA	
**PW2148**	GGCTCCAAGGCCTACGTGAA	qPCR

**PW2123**	CGTCGGAGGGGAAGTTGGTGCC	
**PW2203**	CGTTACCCTGACCACATGAAGCAGCACG ACTTCTTCAAGTCCGCCATGCCTGAAGG CTACGTCCAGGAGCGTACC	qPCR

Table S2. *R. delemar* regulatory sequences identified and used in this study.

H2B1p
AATAAAAAATAGAATTTTACAAATCCTTCTTCTTTCTTTTTTGGTGTAAGCTGTAAAAATTT
TTATACATAAATAAGCGCAAAGATCATTTTTTGGGAAGGGATATCAAATTTTATAAAATATA
AATGCTATTTGAATGTTATAACAATTTAGAGTTCTTGAGAAGCACCGGCCTTCTTGGTCT
TGGTAGGCAACAAAGAGGCATGGATGTTGGGAAGAACACCACCTTGGGCAATAGTGAC
GTGACCGAGAAGCTTGTTTAATTCTTCATCGTTACGGATGGCCAATTGAAGGTGACGAG
GGATGATACGAGACTTCTTGTTATCACGAGCAGCGTTACCAGCCAATTCAAGAATTTCG
GCAGCGAGGTATTCAAGAACAGCAGCAAGATAGACGGGAGCACCAGCACCAACACGT
TGGGCATAGTTACCACGACGAAGAAGACGGTGAATACGACCGACAGGGAATTGAAGAC
CGGCCTTAGAAGAACGAGTTTGAGACTTGGAGTCAGCAGCAGACTTTCCACCAGATTT
TCCACCAGACATTTTTAATAAAGTTTTAAATTAACAAAGAACAAGAAAAAGAAAGAAAAAA
AAAGTTCAAGGCCGCTCTTTATATATGCGTAATCTCCCCTATTCTTTTTTTTTAAAAGACA
GATTTTTCAACCTTTTTTTTTTGAAGTTGAATTTTTTTTTTTCAAAGAATCTCCACAGAAG
GATGGAGGGTAAAAAAAAGATGTAAAAAAATTTTTTTCATGATTTTTTTTAAATCGAAAAA
TCAAAATATAATCATTAGTTTCTGTAGGAAACGCGTTTTTTTCGCGAATTCACGATATATG
GATGTGTTTTTTCAGTCATGTTTTTTTTCGAGAAAAAAAAAGCAAGAAATAAAATTTCCTT
TTTTGGAGTACGAGTCACAGCTGAGAATTTTGTATATAAAACCATTCGATTTTCGAGGAA
CTTTTCGAGTTTTTTTTTGTATTAAAATTTATA

H3p
TTTCTGTGACGCTTAGCACCACCCTTTCCGAGACCTTTACCACCTTTTCCACGACCAGT
CATTTTTAGTTGTATAAATTGAAAAAGAAAAAGAAAAGTGAAAACATGATTCCCTTTTTAT
ATTTTGGGTCAAGTCACACAGCAATCATATTAAATTAGGACACCGTGTTTTTTTTTTTAAA
AAAAAAAAACATTTAAAAATTTCACGGTTAGTAAAAAAATTGCGCGAAAGATTTTTTAAGA
AAAAAAAAAAATGCAAAGAACATTTGTATTGATTTTTATAAACTGAATATATTCAATCATTTA
TTCTAAAAAAAACTTGTTTGTTAGCTAAAAAGTTACTGTCTTAAATAGCTCTACATGTTTAA
TTGTTGGAAGCAACAATTTCGAAGAGAAAAGTGGTTCGTTTCTTTTTTTTTGTTTTTTTTT
TCTCCTTTTTTCGTTCATGAGCTATTATTTTTTTTAAAAAAAATCACAACGCGAAATTTTTT
TAAAAAAAAAGATAGCAGATTCCGACCCATTTTGGAAAAAAGGTTTTGTTTTTTTTTATTA
GTTCTTTTTAATGTCCGGTAGCGTCCCTATAAATAAAGAAGCATGTTCAAATTTTCTACTT
TTCTTTTTCTTTTTTTCTATATCAAACATTAAATAAAT

ADH1p
GGGAAAAAAAAGAAAGAGAATTGGTTAATGAAATAGGAGAGGATGAGTCAAAATATAATT
TACATAAAATTTCTCTTTTTTGTGTTAAATATAATCTAATAGCAGGGGTTTTCTTAGTTTAC

GTTTATATCAAAGTTATCAAGCATACACTTTTTTATGATTTTTCATACTTTAATCCCTTTTAG
TATTCTATTGTTTGAAAGGAGAGAAAAAACAGCTGAGGGTACGGTGCACACGAGATCTT
ACGATAATTTTCCTGCCCAACAGGAAAGAAGTAATTGATCTTGATTGACGCTCGGAGTT
TGCACGTTCGGAGTTTGCACTTCACATTGAGTTATACTCTTACTTATTTTGAAGGAAGGG
ACGAGAAAAGATGTAAATATAATAATAACAGTAGTAAATAGTATGCGCATCAAGAACAGCT
ACCAACAAAAGAGAGAAATATGAGCTTAATAATGAACAATGTAAATGGCAGAATGAAATT
TAATTATCAAAGCGGCATCTTTCAGACCTTCCGTTACTTCCGATAGAGTTTTTTATGCAAA
GTAATAACAACTGTATATATAAAAAAAAGAAGGTTATCAAGCAAAAGCCACAATGTCATAT
CTGGAATAATCAAGAGTAACTATTGAATGTTGGTAGCCAAAAGAGGCACGTAATTTTATG
ACGAAATATCACACAAAAAGATTATTTTGACAATTCATGAATAGGACAGAGATACACCCTA
AACATGAAATGTAAGCTATATTTAAACACCTCAAGTTAATTTTGAAGCTTCATTTGTATTAT
TGTAACCATTTAGACAAGCTAAATCCTTTTTATTATTGTCCTTATTGATTTTATCCAGATTA
CCGTATCTAAAGAGCGATCAACAGAAAAACGGCTGATTTTAGACCAAAGTTTCACAAAC
TACATTTGCATGAACGTCATATATATATAAACCTTGACTTTTCTTTTTTTTTTTTTTTTTTTT
TTCATCATCAATTAATACAAATAAAAAACCAA

TEF1p

AATTGTGTGAAAGTGTAAATTCCATAATAAACAGCGAAATCAAGACAGATCAAAACTCCT
GAAGTACTTGAATCACAAACATACCCTTGGAAACACGTATCATATTTATGTATACTGAAAG
GCACTTGAATAGCTCAAAAGGTATTTGTTACCGCCATTCATTAAATTAAATTTTGACCAAA
ACGATGCTAAAAAATTTTTTACCTTTCGTTCCTGAAAATTTTTTAGAGGTAACCAATAGCA
GACTATTCCGAAATTGAATTGACTAGAGAAAAACGGCGTTTATGACAGCAAAGTAGTTTA
TAACCAAGTCTAGTGTTATTTGATATTAAACCAATTTTTTCCATATTGGTTTTTTAAACTATA
GTTCTCATTCGTTACAATGATAAAGACTACCTTGGCGGAGTAAAAAGAAAAAGCAAAAC
AGAGAGTCTCCGAACGAAATAGAAACATTTCGAAAATAGTTTGCTTCACTTCAAATAACA
GTAAACATCAAGCAAACATTGTGGCTACCATACATTGCTCTTTTTGGTATCTATTTCAACG
TGTGAGAATGAACAAGAAAGAAAAAAGTAAAAGAGCAAAAAAGAGTAATTCCTACAGTA
AAACTTTTGTTGTGCAACCCAATCAAGTCATGCCGTTGTGACCATTTCCGCGGTGGCTT
TTCACGCGGAACAAGAAAAAAAAATTTTAAAACGAGAAATTTTTTTGTTATTGTTTGTTTTC
CCTTTTTTCTGTTACTTTATAAACTTCCTCAAGTAAGGATACTCACTACGCCATCCAACAT
GTACTAATTCTATTCATCATAGCATGGCT

TEF2p

TAAACCAATTCAAAATTTATTTCAATACTGCATCACTCACACAAAAGAATTTATGCAAAAC
TAACAACTTGGCTTACTACGATGTTTGGACCACATTTACAGTACTAGAATCTGCTAAGCA
TGCTACTGCTACTATGCCAATAGCAATTAAAAGAGGATATTCCACTTTGTGTTTCGCTGAT
GCTGCGGCTCAATTCTTATTTGAGCCTCAATTCAAAAGCTCACTTGTTCGAGATGTTCA
ATTCTTGAACAGTTCATGTTTTGTAAAAGATTCAAATAGTACATTTTTATTCTTCTATTTGC
ACTGAACCCACCAAGTCCTGATGATAATGTCTTTCAACTCAACATCGACGCGACAAAAG
CTCAAGCAAAAGCAAATCTCTCGATTCTTTCCCTTTCCTTTATTTCTTTTCTATCCATTTG
TGGATAGAAAAAATTATTACAACTTTATTCAACAATCCATGGCCCGCTTTTAGTTTAGCAA
CACTAACTTGTTTTTTTTTCGTATAGAAAAGAAAGAAGTCAAATCAAATAACAACCCCGCG
GCCAAATCAGCAGTCAAACCAGCGGCCAAACCAGCGACCAAACCAACCGCTAAACCA
ACAGTGAAAAAATCAACAGGAGATGAAGTAGAGTTTTTGTCTGATGAAGAAGAACTTGA
TCGAGAT

PDCAt

TCTAGACTAGAGTCGACCTGCAGGAGGATCCAATCTTAGAATTCATCTTTTTTTGTATCA
TTCGTTCTATACTTCATTACTACAGTAAATTCATAATAAAATCATATTTTTGAAAATAAGCTT
TTTTTTTCTTTTACCTGTGGGCCCTATGATGATTGGTGTAACTTTTACAAGCTTTGGATTG
CCAAGTTTTATCAGCATGTGACAAGTCAGCACACGCTCTTGAATATTTCTAGGCAACAA
GGCTTTGATTTCTTTTAGGGACTTGGTTTATATTGAATCCTTTTAAGGATTGTATTTAAAAA
TAGTTTTATTGCAAGAAATGATGTATGTGTGGGTATTTGTTACAATTTAATTAATAAGGGC
GAATTCCAAGCTTGGCGTAATCA

Figure S1

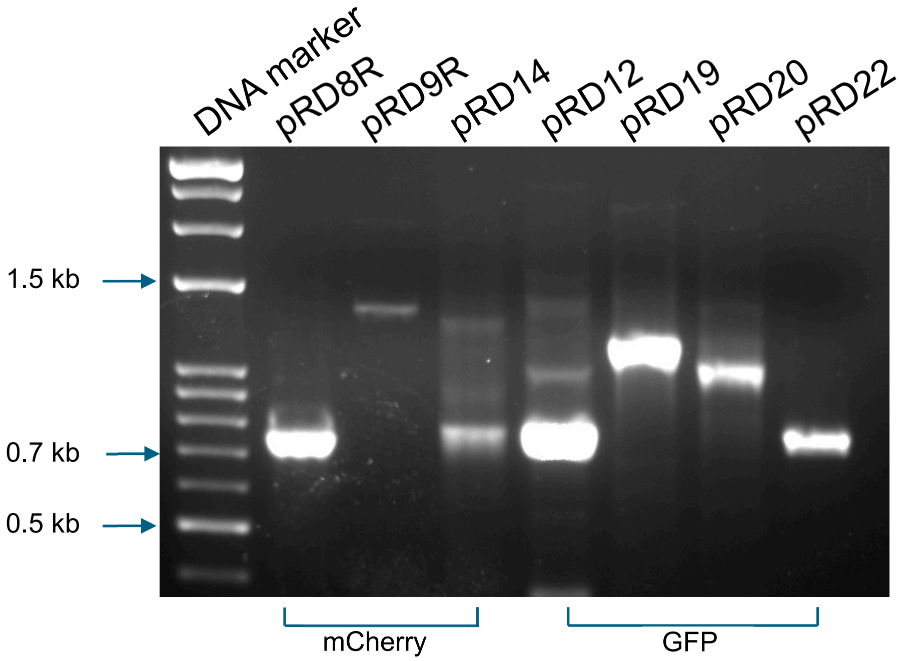

Supplement: Supplementary file 1 [file jof-12-00771-s001.zip › jof-4608794-supplementary.pdf]
